# Persistent Tissue Kinetics and Redistribution of Nanoparticles, Quantum Dot 705, in Mice: ICP-MS Quantitative Assessment

**DOI:** 10.1289/ehp.10290

**Published:** 2007-06-14

**Authors:** Raymond S.H. Yang, Louis W. Chang, Jui-Pin Wu, Ming-Hsien Tsai, Hsiu-Jen Wang, Yu-Chun Kuo, Teng-Kuang Yeh, Chung Shi Yang, Pinpin Lin

**Affiliations:** 1 Division of Environmental Health and Occupational Medicine, National Health Research Institutes, Zhunan, Taiwan; 2 Quantitative and Computational Toxicology Group, Department of Environmental and Radiological Health Sciences, Colorado State University, Fort Collins, Colorado, USA; 3 Division of Biotechnology and Pharmaceutical Research, National Health Research Institutes, Zhunan, Taiwan; 4 Center for Nanomedicine Research, National Health Research Institutes, Zhunan, Taiwan

**Keywords:** ICP-MS, kinetics, nanoparticles, quantum dot 705, redistribution, mice

## Abstract

**Background:**

Quantum dots (QDs) are autofluorescent semiconductor nanocrystals that can be used for *in vivo* biomedical imaging. However, we know little about their *in vivo* disposition and health consequences.

**Objectives:**

We assessed the tissue disposition and pharmacokinetics of QD705 in mice.

**Methods:**

We determined quantitatively the blood and tissue kinetics of QD705 in mice after single intravenous (iv) injection at the dose of 40 pmol for up to 28 days. Inductively coupled plasma–mass spectrometry (ICP-MS) measurement of cadmium was the primary method of quantification of QD705. Fluorescence light microscopy revealed the localization of QD705 in tissues.

**Results:**

Plasma half-life of QD705 in mice was short (18.5 hr), but ICP-MS analyses revealed QD705 persisted and even continued to increase in the spleen, liver, and kidney 28 days after an iv dose. Considerable time-dependent redistribution from body mass to liver and kidney was apparent between 1 and 28 days postdosing. The recoveries at both time points were near 100%; all QD705s reside in the body. Neither fecal nor urinary excretion of QD705 was detected appreciably in 28 days postdosing. Fluorescence microscopy demonstrated deposition of QD705 in the liver, spleen, and kidneys.

**Conclusion:**

Judging from the continued increase in the liver (29–42% of the administered dose), kidney (1.5–9.2%), and spleen (4.8–5.2%) between 1 and 28 days without any appreciable excretion, QD705 has a very long half-life, potentially weeks or even months, in the body and its health consequences deserve serious consideration.

Quantum dots (QDs) are semiconductor nanocrystals approximately 2–100 nm in diameter. Structurally, they have a metalloid core and a cap or shell (Dabbousi et al. 1997). Organic coatings can be added to the surface to achieve bioavailability or targeting toward specific sites. QDs possess unique luminescent properties (Akerman et al. 2002). One probable use of QDs is as optimal fluorophores for *in vivo* biomedical imaging (Larson et al. 2003; Morgan et al. 2005). It can also be used to target specific cells (e.g.*,* labeling neoplastic cells) after conjugation with specific bioactive moieties (Cai et al. 2006; Voura et al. 2004). Basically, QD cores consist of various metal complexes. Cadmium–tellurium (CdTe) and cadmium–selenium (CdSe) cores with zinc sulfide (ZnS) shells represent the newest structures synthesized and are most commonly seen in the QD industry (Lim et al. 2003). Our test material, QD705, has a CdTe core with a ZnS shell although analyses in our laboratory revealed 46, 11, and 1% Cd, Se, and Te, respectively. These nanocrystals are surface modified with methoxy-polyethylene glycol (PEG-5000), an inert biologically compatible polymer. Both Cd and Se are well-known metals that are toxic to humans, causing heptatic, renal, neurologic and/or genetic toxicities (Bertin and Averbeck 2006; Klaassen and Liu 1997; Umemura 2000; Vinceti et al. 2001). Te was also reported to cause neuropathy (Widy-Tyszkiewicz et al. 2002). Exposures to these toxic elements are likely when degradation of QD705 occurs *in vivo*.

Despite the immense potential for the medical applications of QDs and the intent of introducing these nanocrystals into the human body, we know little about the disposition [absorption, distribution, metabolism, elimination (ADME)] and health consequences of QDs in animals and humans (Hardman 2006; Nel et al. 2006). Three excellent recent reviews are available on QDs and nanomaterials in general (Hardman 2006; Maysinger et al. 2006; Nel et al. 2006). A commonly agreed issue is the lack of biosafety data for nanomaterials and the need for human health assessment. The toxicity of QDs, if present, depends on a variety of factors related to the physicochemical properties of the quantum dots, as well as the environmental conditions they are in (Hardman 2006). These include the size, charge, concentration, outer coating, and mechanical stability of the QDs (Hardman 2006).

A large proportion of the available literature involves *in vitro* studies on a number of cytotoxic parameters. Although some investigators reported QDs do not appear to cause toxicity, others demonstrated a variety of cytotoxic effects (Chen and Gerion 2004; Hanaki et al. 2003; Hoshino et al. 2004a, 2004b; Jaiswal et al. 2003; Lovric et al. 2005; Shiohara et al. 2004; Voura et al. 2004). A few *in vivo* studies are available in the literature as well. Akerman et al. (2002) demonstrated they were able to target the accumulation of ZnS-capped CdSe QDs in specific tissues by varying the coatings. They also reported that QDs accumulated in both the liver and spleen in addition to the intended target tissues. Dubertret et al. (2002) microinjected QD-micelles into the embryos of the carnivorous frog *Xenopus* and reported that the QDs were stable, nontoxic, and slow to photobleach. Further, they suggested QD’s lack of interference with embryogenesis (Dubertret et al. 2002). Ballou et al. (2004) reported that the circulating half-lives in mice were approximately 12–70 min for QD630, QD645, and QD655. Whole body fluorescence imaging studies indicated that QD is present in liver, lymph nodes, and bone marrow for at least a month after an intravenous (iv) dosing (Ballou et al. 2004). Hoshino et al. (2004b) incorporated QDs into the EL-4 cells, then injected these cells intravenously into the nude mice to study the fate of the EL-4 cell and QD on the basis of fluorescence. These investigators detected QDs in the kidneys, liver, lung, and spleen for up to 7 days without damage or toxicity to the mice (Hoshino et al. 2004b). These pioneering studies elegantly used the fluorescence properties of QDs and provided valuable information regarding the fate of QDs in the whole animals. As fluorescence intensity depends on the microenvironment, studies based on fluorescence are usually qualitative in nature. Thus, quantitative pharmacokinetic analyses of QDs in the animals are needed. In a recent publication, which is not easily searchable in biomedical databases, Fischer et al. (2006) reported pharmacokinetics and tissue sequestration and distribution in male Sprague-Dawley rats of two QDs synthesized in the authors’ own laboratories (Fischer et al. 2006). Half-lives were determined to be 39–59 min and the QDs were cleared from the plasma between 0.59 and 1.23 mL/min/kg (Fischer et al. 2006). The liver took up 40 to 90% of the QDs after 90 min, whereas the other tissues retained only a small amounts (Fischer et al. 2006).

In our present study, we conducted three types of experiments on a commercially available quantum dot, QD705: *a*) we determined quantitatively the blood and tissue kinetics of QD705 in mice after single iv injection for up to 28 days; *b*) we performed two mass balance studies at 1 and 28 days after a single iv dosing to see if we may recover quantitatively the administered dose and to discern the distribution of QD705; and *c*) we localized QD705 in major target organs using fluorescence microscopy.

## Materials and Methods

The nanoparticles, QD705, used in our experiments are commercially available from Quantum Dot Corporation (Hayward, CA, USA) as Qtracker 705 nontargeted quantum dots. Each particle has a CdTe core and ZnS shell with methoxy-PEG-5000 coating. The diameter is about 13 nm, and the molecular weight is 1.5 × 10^6^ g/mol. According to Quantum Dot Corporation Certificate of Analysis, 30 November 2005, and Giepmans et al. (2005), fluorescence of QD705 ranges from 650 to 750 nm with an emission maximum around 700–715 nm. Each tube of this product contained 200 μL of a 2-μM solution in 50 mM borate buffer, pH 8.3. We purchased 6-week-old male ICR mice from BioLASCO (Taiwan) and acclimated the mice for 2 weeks in the animal facilities at the National Health Research Institutes (NHRI). All animal treatments and experimental protocol for this study were subjected to the review and approval of the Animal Control Committee at NHRI. Thus, animal handling was in accordance with standard animal husbandry practice and regulation; animals were treated humanely and with regard for alleviation of suffering throughout the study. All mice in this study were under 12-hr light/dark cycle, 23 ± 1°C, 39–43% relative humidity; water and food were available *ad libitum*. At the start of the experiments, the mice were approximately 8 weeks old and weighed between 32.9 and 38.7 g each. We selected six mice/time point, randomly, for time-course studies. We injected each mouse, via tail veins, with 40 pmol QD705 (20 μl of a 2-μM solution) in saline; the injection volume was 100 μL/mouse. We chose the iv route to mimic potential human medical imaging application and provide cleaner pharmacokinetic profiles without the complication of the absorption phase. Serial sacrifices (under pentobarbital anesthesia) took place at 1, 4, 24 hr at 3, 7, 14, and 28 days after dosing of QD705. At each of these sacrifices, we measured and recorded body weights as well as organ weights of each animal. Tissue samples collected at each time point included plasma, red blood cells (RBCs), liver, lungs, kidneys, spleen, muscle, thymus, fat, brain, skin (ear) and bones. Subsequently, we analyzed and quantified QD705 in these tissues. To anticipate possible further analyses, we saved the carcass individually with proper identification. QD705 analyses were performed on the basis of quantification of ^111^Cd concentrations in various tissues using inductively coupled plasma–mass spectrometry (ICP-MS) (Elan6100; PerkinElmer, Shelton, CT, USA). Briefly, we dried the tissues at 95 ± 2°C for 16 hr. They were then weighed and liquefied with 5 mL nitric acid and 1 mL hydrogen peroxide and underwent microwave digestion (Multiwave 3000; Anton Paar GmbH, Graz, Austria). ICP-MS measurement of Cd (mass = *m/z* 111 and 114) was the primary method of quantification of QD705 in tissues. With plasma samples, however, fluorescence spectroscopy was an additional method for quantification of QD705 (Ballou et al. 2004). With the fluorescence method, we centrifuged the heparinized whole blood samples at 1,500 × *g* (Allegra X-22R centrifuge; Beckman Coulter, Fullerton, CA, USA) for 15 min at 4°C to isolate the plasma. Fluorescence spectroscopic analyses provided fluorescence intensities (FIs) (excitation/emission: 500/705 nm) of 20% plasma samples and Rhodamine 101 (a positive control). Normalization of these results with the absorbance at 705 nm was necessary. We then calculated the ratio for each plasma sample from the FI/A ratio of sample over the FI/absorbance ratio of Rhodamine 101 (Ballou et al. 2004). For establishing standard curves, we used a range of concentrations (0.1, 0.2, 0.4, 0.8, 1.6, 3.2, and 6.4 nM) of QD705 in 20% control plasma in saline or normal saline. Comparison of these standard curves was essential to assess the quenching effect of plasma on fluorescence intensity. Samples, in duplicate, were placed in block 96-well (100 μL/well) for measuring fluorescence intensity as well as absorbance. The pharmacokinetic parameters were analyzed from Cd time-course plasma concentrations by using WinNonLin software program (version 3.1; Pharsight, Mountain View, CA, USA).

## Results

**Figure 1:**
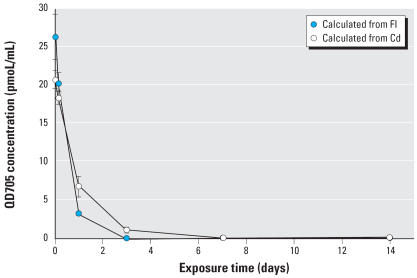
Plasma kinetics of QD705 in ICR mice after iv dosing. ICR mice were injected via tail vein with 40 pmol QD705. QD concentrations in the plasma were calculated from fluorescene intensity (FI) and Cd concentrations at 1, 4, 24 hr at 3, 7, and 14 days after dosing of QD705. The 28-day data (close to zero) are not included to provide a better plot to show the initial phase. The data were mean ± SD of six replicates.

Figure 1 provides the comparative results of plasma kinetics of QD705 in mice based on ICP-MS analyses for ^111^Cd and florescence spectroscopy. As shown in Figure 1, the time-course curves based on two different analytical methods are comparable. The plasma kinetics of QD705 (Figure 1) revealed that its clearance from the blood was 2.3 mL/hr/kg. In about a week, plasma QD705 level was down to the background level. From the plasma Cd concentration versus time data, we calculated the plasma half-life to be 18.5 hr. QD705 did not appear to have any specific affinity toward RBCs.

**Figure 2:**
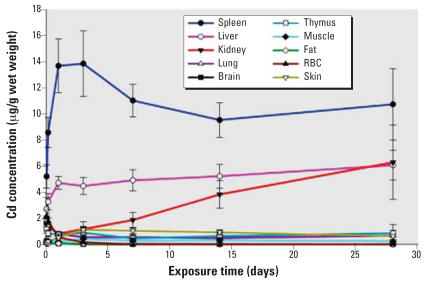
Tissue kinetics of QD705 in ICR mice after iv dosing. ICR mice were injected via tail vein with 40 pmol QD705. Serial sacrifices were carried out at 1, 4, 24 hr at 3, 7, 14, and 28 days after dosing. Several organs/tissues, including spleen, liver, kidney, lung, brain, thymus, muscle, bone, fat, RBCs, and skin were isolated to determine Cd concentrations with ICP-MS. The data were mean ± SD of six replicates.

Initial attempts of tissue analyses of QD705 using fluorescence spectroscopy were unsuccessful because of problems related to extraction of QD705 from the tissues and the interference of fluorescence detection by endogenous substances in the tissues. Thus, all subsequent tissue analyses were based on ICP-MS analyses of Cd. Contrary to its relatively transient fate in the plasma, high levels of QD705 persisted in the spleen, liver, and kidneys throughout the experimental period (Figure 2). In the liver and kidneys (Figure 2), there was even a tendency of increasing tissue concentration with time. It is quite apparent that the overall half-life of QD705 in the body is very long, possibly in terms of weeks if not months.

**Figure 3:**
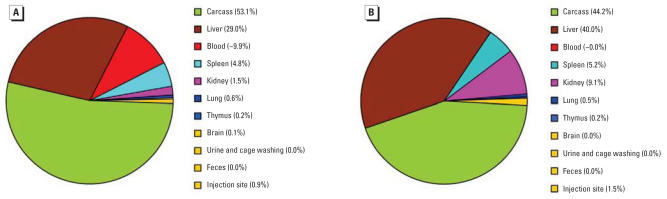
Mass balance studies of an iv dose of QD705 in ICR mice. Mass balance studies were carried out at 1 day (*A*) and 28 days (*B*) after dosing. Overall recoveries for 1 day and 28 days were, respectively, 102.2 and 108.8%.

We placed each group of six mice for the 24-hr and 28-day sacrifice time points in metabolism cages (two per cage) and performed mass balance studies. The purpose was to examine the recovery rate of the iv dose and to calculate the proportion of QD705 distribution in the plasma, RBCs, liver, spleen, kidney, lung, muscle, skin, thymus, fat, brain, bone, carcass, urine, and feces. Because muscle, skin, and bones contained very low levels of QD705, we combined their levels into that in carcass. We chose to perform mass balance studies at the beginning (1 day) and the end (28 days) of the experimental duration to assess the persistence of QD705 in the body. As shown in Figure 3A and B, we were able to recover 100% of an iv dose of QD705 from the body at both the 1-and 28-day time points, indicating very little excretion during the 28 days. Forty-four to 50% of QD705 retained in carcass mainly distributed in the large masses of muscle, skin, and bone. QD705 also accumulated in the liver (29.0–40.0%), spleen (4.8–5.2%), and kidney (1.5–9.1%). There was an obvious increase with time in the concentration of QD705 in kidneys; a similar trend was apparent in the liver although the rate of increase was not as rapid. It is particularly interesting to note the time-dependent tissue redistribution into the liver and kidneys, from tissue masses in the carcass, between 1 day and 28 days (Figure 3A and B). However, QD705 was not detectable in feces and barely detectable in the urine (0.01–0.04%). The average recovery rate of the iv dose of QD705 was 102.2 ± 20.5% in the 1-day mass balance study and 108.8 ± 6.1% in the 28-day mass balance study. These results indicated that we accounted for all the QD705 injected intravenously into the mice. It is important to emphasize that essentially no excretion occurred in 28 days.

**Figure 4:**
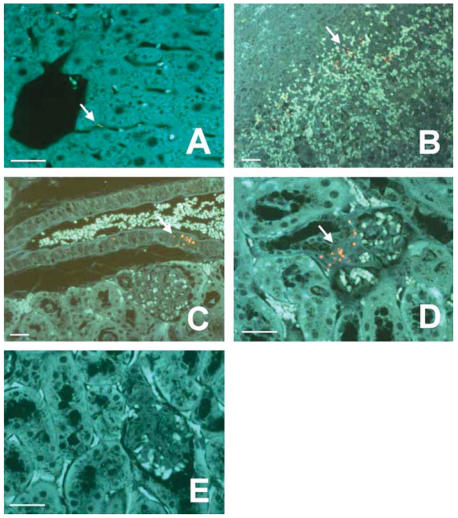
Distribution and localization of QD705 in various tissues in mice 28 days following an iv dose. QD705 was detected as red fluorescent particles against the blue–green autofluorescent background (tissues) under the fluorescence microscope. (*A*) Liver. A few red fluorescent particles were seen in the sinusoids; no significant amount of QD705 was found in the hepatocytes. (*B*) Spleen. Abundant QD705 was located in the splenic tissue. Most of the red fluorescent particles (QD705) were found in the red pulps. (*C* and *D*) Kidney. Such red fluorescent particles were not observed in control kidney tissues (*E*) showing that the red fluorescent particles were QD related. Foci of QD705 (collections of red fluorescent particles) could be identified in the large renal vessel and in the glomerular-associated vessels. Scale bar = 50 mm.

To assess the localization of QD705 in the liver, spleen, and kidney, we took advantage of the fluorescence property of QD705. These tissues underwent the standard histologic processing of fixation, embedding in paraffin, and microsection. We localized QD705 in these tissue sections with the aid of a fluorescence microscopy (excitation: 350–380 nm/emission ≥ 400 nm). Pathological examination revealed marked sinusoidal congestion and increased multinucleated giant cells in red pulps regions (vascular areas) of the spleen. Liver and kidneys displayed no remarkable abnormalities both grossly and microscopically. Tissue localization of QD705 appeared as fine red fluorescent granules under the fluorescence microscope (Figure 4). Such localizations were especially prominent at the linings of hepatic sinusoids (Figure 4A), red pulps of spleen (Figure 4B), renal vessels, and glomerular vasculature in the kidneys (Figure 4C and D). Thus, the accumulation of QD705 in tissues occurred consistently in vascular-rich areas.

## Discussion

In assessing the toxic potential of nanomaterials in general, Nel et al. (2006) excellently summarized the potential biological and bio-kinetic properties of nanomaterials in relation to their physicochemical properties. These investigators pointed out that the biological impacts of nanomaterials are very much a function of size, chemical composition, surface structure, solubility, shape, and aggregation (Nel et al. 2006). Based on the physicochemical properties of nanomaterials, as well as their interactions with other environmental factors such as ultraviolet (UV) activation, Nel et al. (2006) deduced possible mechanisms by which nanomaterials interact with biological tissues. These include *a*) discontinuous crystal planes and defects of the nanomaterials generate active electronic configurations for electron donors/acceptors; *b*) coating metals and organics create redox cycling and catalytic chemistry; *c*) UV activation of electron-hole pairs leads to bond splitting and radical formation; and *d*) particle dissolution, coating, passivation, and hydrophobicity/hydrophilicity can cause interactions with cell membranes, cells, and surrounding media (Nel et al. 2006). Maysinger et al. (2006), on the other hand, suggested that internalization of QDs by cells could be achieved by ligand–receptor or antibody-mediated entry, endocytosis, electroporation, and oxidative damage of plasma membrane. Whether any of these mechanisms leads to the accumulation and redistribution of QD705 in the spleen, liver, and kidneys in the present study is not clear. We plan to explore this further through additional studies in our laboratories.

Tissue localizations of QD705 were mainly at the linings of hepatic sinusoids, red pulps of spleen, renal vessels, and glomerular vasculature in the kidneys. Our observations have the following implications: *a*) the visualization of red fluorescence (Figure 4A–C) suggest aggregation of a large number of QD705 nanocrystals *in situ* because fluorescence from one or a few particles of QD705 would have been invisible under the microscope; *b*) a major mechanism of disposition of QD705 involves mononuclear phagocytic system and organs. This observation is interesting because Akerman et al. (2002) indicated that PEG coatings on QDs were adsorption resistant and minimized recognition by the mononuclear phagocytic system; and *c*) the congregation of QD705 in the renal vessels (Figure 4C and D) and the continuing increase of kidney concentration (Figure 2) would lead to further accumulation in the glomeruli which, in turn, would cause eventual renal toxicity. Similar increases and accumulation with time in the liver would similarly induce hepatotoxicity.

In conclusion, we have presented quantitative time-course results from long-term pharmacokinetic and mass balance studies of QD705 in a common laboratory animal. Initial findings revealed the persistent nature of QD705 with regard to tissue kinetics. We anticipate a very long biological half-life, possibly in weeks or months, of QD705 nanocrystals in animals and humans. Such a long-term accumulation in the vital organs will most likely result in health consequences. Because these nanocrystals are intended for human medical use, thorough safety evaluation needs to be carried out. Using this perspective, we are expanding our pharmacokinetic studies to include more thorough plasma kinetic studies with classic pharmacokinetic analyses. We are also constructing a physiologically based pharmacokinetic model for QD705 in mice on the basis of all the tissue kinetic data and other available information. Similarly, parallel toxicology studies including ultrastructural examinations are in progress. From a totally different perspective, those chemistry colleagues who are actively engaging in the synthesis and formulation of QDs may wish to look into possible modifications on QD705 or similar nanocrystals such that enhanced excretion by animals, including humans, can be achieved to optimize the utility of a potentially very useful lines of nanoproducts.
